# Insights into the Fluxional Processes of Monomethylcyclohexenyl Manganese Tricarbonyl

**DOI:** 10.3390/molecules28073232

**Published:** 2023-04-04

**Authors:** Guangchao Liang, Min Zhang

**Affiliations:** 1 Academy of Advanced Interdisciplinary Research, Xidian University, Xi’an 710071, China; 2 Department of Pharmacy, School of Medicine, Xi’an International University, Xi’an 710077, China; zhangmin01@xaiu.edu.cn

**Keywords:** agostic interaction, fluxionality, VT-NMR, density functional theory

## Abstract

Multiple fluxional processes of 6-monomethylcyclohexenylmanganese tricarbonyl [(6-MeC_6_H_8_)Mn(CO)_3_, complex **1**] and 5-monomethylcyclohexenylmanganese tricarbonyl [(5-MeC_6_H_8_)Mn(CO)_3_, complex **2**] have been explored using density functional theory (DFT) computations. The contributions of four agostomers—**1**, **2**, **3,** and **4**—to the (MeC_6_H_8_)Mn(CO)_3_ exchange processes were revealed. The computational results demonstrated that the 1, 2-agostic isomerization only occurred via the η^4^-diene hydride transition state (**TS-1-2**, 14.0 kcal/mol), which is consistent with the experimentally proposed high-energy exchange process (16.0 kcal/mol). Excellent agreement is observed (R^2^ = 0.9862) when comparing the computed and experimentally observed variable temperature ^1^H NMR chemical shifts. With these results, important insights into the role of agostic interaction in the homogeneous catalysis process could be made, especially with regard to transition metal catalyzed C-H activation.

## 1. Introduction

**Scheme 1:**
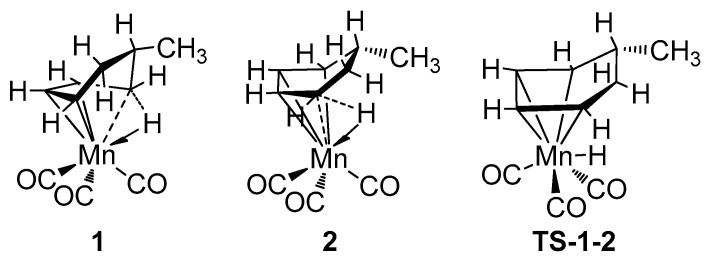
Structures of 6-monomethyl, 5-monomethyl isomers of (MeC_6_H_8_)Mn(CO)_3_ and the proposed transition state.

Unlike exo-methyl products from the protonation of the double hydride addition to the (η^6^-methylbenzene)manganese tricarbonyl cation [(η^6^-MeC_6_H_5_)Mn(CO)_3_^+^] [1], the reaction between (η^4^-1, 3-cyclohexadiene)manganese tricarbonyl anion [(η^4^-C_6_H_8_)Mn(CO)_3_^–^] and excess CH_3_I yielded two different endo-methyl products: 6-monomethylcyclohexenylmanganese tricarbonyl [(6-MeC_6_H_8_)Mn(CO)_3_, complex **1**, half-chair conformation, Scheme 1]; and the 5-monomethyl analogue [(5-MeC_6_H_8_)Mn(CO)_3_, complex **2**, half-chair conformation, Scheme 1] [2,3,4]. Experimental and computational studies of the unsubstituted parent analogue cyclohexenyl manganese tricarbonyl [(C_6_H_9_)Mn(CO)_3_] [2,5] suggested that 6-monomethyl (complex **1**) and 5-monomethyl isomers (complex **2**) could also undergo several fluxional processes. However, the complexities in the obtained variable temperature ^1^H NMR spectra limited the discrimination of all possible exchange processes of (MeC_6_H_8_)Mn(CO)_3_, and only two processes could be estimated from the experimentally obtained ^1^H NMR spectra: (1) a low-energy process estimated as 8.3 kcal/mol, proceeding through the fast endo C-H exchange; and (2) a high-energy exchange process estimated as 16.0 kcal/mol, proceeding through the diene hydride species [1]. These values are quite similar to the parent system cyclohexenyl manganese tricarbonyl (C_6_H_9_)Mn(CO)_3_ (8.3 kcal/mol and 15.4 kcal/mol) [2]. Variable temperature ^1^H NMR spectra suggested the conversion between the major isomer (complex **1**, 78%) and minor isomer (complex **2**, 22%) through the η^4^-diene hydride transition state (**TS-1-2**, Scheme 1) [4]. This asymmetrical transition state caused by a substituted methyl group, in contrast to the C*_s_* symmetrical η^4^-diene hydride transition state of the unsubstituted parent analogue [(C_6_H_9_)Mn(CO)_3_], together with other possible transition states, made it inconvenient to assign resonances in the variable temperature ^1^H NMR spectra.

**Scheme 2:**
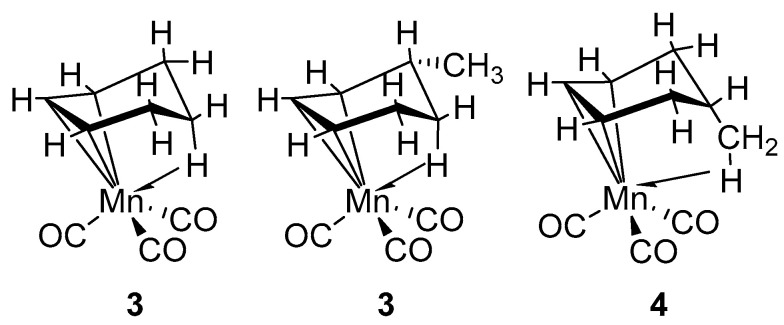
Possible agostomers (**3**, **4**) of (MeC_6_H_8_)Mn(CO)_3_ and the related analogue (**3**).

Several ^1^H NMR resonances of complex **1** and **2**, such as 6H_exo_ of complex **1** and **2** and 3H of complex **2**, could not be properly assigned experimentally due to the spectral complexities [4]. The experimental ^1^H NMR resonances indicated that the 5H_exo_ (0.4 ppm) of complex **2** was quite different from the 5H_exo_ (2.2 ppm) and 6H_exo_ (1.2 ppm) of 5, 6-dimethyl analogue [5, 6-dimethyl cyclohexenyl manganese tricarbonyl, (5, 6-Me_2_C_6_H_7_)Mn(CO)_3_]. The proton chemical shift of the 6-methyl group (0.1 ppm) of complex **1** was also inconsistent with that of the 5-methyl group (0.6 ppm) of complex **2**, 5-methyl (0.8 ppm), and 6-methyl (0.7 ppm) of the 5, 6-dimethyl analogue [1,4]. These inconsistencies raised the question of how the accurate ^1^H NMR spectra of these complexes with multiple exchange processes could be found. Previous results demonstrated the important role of the chair agostomer with weak agostic interaction (complex **3**, Scheme 2) in the fluxionality of the unsubstituted analogue [(C_6_H_9_)Mn(CO)_3_] [5]. Two possible chair agostomers (complexes **3** and **4**, Scheme 2) could also exist during the conversion between 6-monomethyl (complex **1**) and 5-monomethyl systems (complex **2**). However, some difficulty was encountered in experiments to clarify the nature of the M-H-C bond (agostic or anagostic interaction) of the second chair conformation (complex **4**), which is caused by the effect of the substituted methyl group.

The purposes of this study are to understand the fluxionalities of the monomethyl cyclohexenyl manganese tricarbonyl [(MeC_6_H_8_)Mn(CO)_3_, complex **1** and **2**], to accurately characterize the Mn-H-C agostic interaction for the isomers, to correctly investigate these multiple exchange processes of (MeC_6_H_8_)Mn(CO)_3_, and to computationally simulate the accurate ^1^H NMR spectra of these exchange processes. The results could potentially establish insights into the role of Mn-H-C agostic interaction in the homogeneous catalysis [6,7,8].

## 2. Results and Discussion

### 2.1. Structure and Bonding

**Figure 1:**
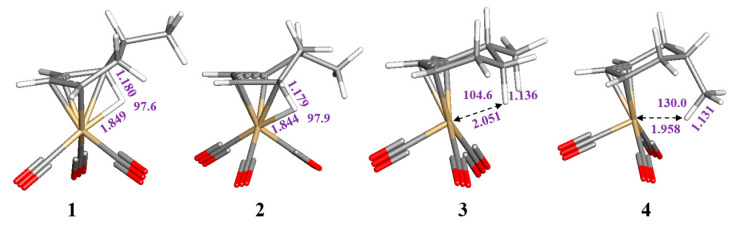
DFT optimized structures of intermediate and transition state (bottom, left to right). Color code: yellow, Mn; red, O; gray, C; white, H. Selected atom distances are given in Å and bond angles are in °.

The PBEPBE/BS1-Auto optimized gas-phase structures of 6-monomethyl isomer (complex **1**, Figure 1) and 5-monomethyl isomer (complex **2**, Figure 1) showed they were half-chair conforms. The computationally optimized 6-monomethyl isomer (complex **1**, Figure 1) matched well with the reported X-ray crystal structure (CSD entry: BEZYEK) [2], and the RMSD (root mean square deviation) for atoms without H was 0.037 Å (Table S1). A negligible effect of dispersion correction on the optimized gas-phase structure was observed, and the RMSD (root mean square deviation) for PBEPBE-D3(BJ)/BS1-Auto was 0.034 Å (Table S1). The agostic bonding characters [9,10], such as the M-H bond length, the C-H bond length, the proton chemical shifts, and the spin coupling constants (J_CH_) of the optimized structures of complexes **1**, **2**, and two other complexes **3**, **4** are listed in Table 1.

**Figure 2:**
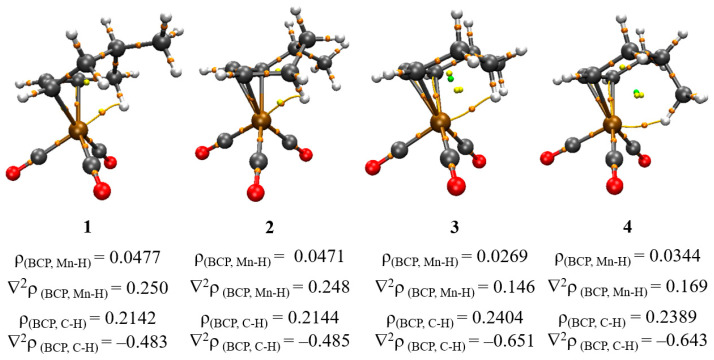
The AIM (atoms-in-molecules) analysis of the optimized agostomers **1**, **2**, **3**, and **4**. The orange balls represent the BCP (bond critical point), the yellow balls represent the RCP (ring critical point), the green balls represent the CCP (cage critical point), and the bond paths are shown in orange. Atom color codes: C, gray; H, white; O, red; N, blue; P, orange; Mo, cyan; Mn, ochre; Rh, purple. The electron densities of bond critical points [ρ_(BCP)_] and the Laplacian of the electron density (∇^2^ρ) are given in a.u.

The prolonged C-H bond length (H_endo_ vs. H_exo_), and the upfield proton chemical shifts (H_endo_ vs. H_exo_) were all observed for complexes **1**, **2**, **3**, and **4** (Table 1), which clearly demonstrated the existence of the Mn-C-H agostic interaction [9,11]. The Mn-C-H bond angle in complex **1** is 97.6°, which is notable for being slightly smaller than that of complex **2** (97.9°). The Mn-H bond distance in complex **1** is 1.849 Å, which is also slightly longer than that of complex **2** (1.844 Å). The observed Mn-C-H bond angles and Mn-H bond distances in complexes **1** and **2** demonstrated that the strengths of the Mn-C-H agostic interaction in complexes **1** and **2** are equivalent. This is also supported by the AIM (atoms-in-molecules) analysis of the optimized complexes **1** and **2** (Figure 2). The electron density of the Mn-H bond critical points (ρ_BCP_) in complexes **1** and **2** are 0.0477 and 0.0471, respectively. Negligible differences of the electron density of C-H_(endo)_ bond critical points (ρ_BCP_) in complexes **1** (0.2142, Figure 2) and **2** (0.2144, Figure 2) were also observed. The Mn-C-H bond angle in complexes **3** and **4** are 104.6° and 130.0°, respectively, which are significantly bigger that those in complexes **1** (97.6°, Table 1) and **2** (97.9°, Table 1). The C-H_(endo)_ bond distance in complexes **3** and **4** are 1.136 Å and 1.131 Å, respectively, which are considerably shorter that those in complexes **1** (1.180 Å, Table 1) and **2** (1.179 Å, Table 1). The relatively stronger C-H_(endo)_ bonds in complexes **3** and **4** are also verified by the higher electron density of C-H_(endo)_ bond critical points, and ρ_(BCP, C-H)_ for C-H_(endo)_ bond in complexes **3** and **4** are 0.2404 and 0.2389, respectively. A weak Mn-C-H agostic interaction is characterized by a strong C-H_(endo)_ bond and a weak Mn-C-H bond [6], and complex **3** has a much weaker Mn-C-H agostic interaction than complex **4**. The Laplacian of the electron density (∇^2^ρ) is utilized in classifying the covalent interaction (locally concentrated with ∇^2^ρ < 0) and the non-covalent interaction (locally depleted with ∇^2^ρ > 0) [12,13]. With regard to the C-H BCPs, the ∇^2^ρ is evidently negative, which strongly demonstrates the covalent nature of the C-H bonds (Figure 2). The ionic Mn-H interaction is demonstrated by the positive ∇^2^ρ the Mn-H BCPs. The absolute values of ∇^2^ρ correspond to the strengths of the covalent and non-covalent interaction, and the order of C-H is **3** > **4** > **2** ~ **1**. The observed values of the Laplacian of the electron density (∇^2^ρ) of the Mn-H and C-H BCPs are consistent with the relative strengths of Mn-C-H agostic interaction in complexes **1**, **2**, **3**, and **4**. Based on the above discussions, complexes **1** and **2** could be assigned as the classical agostomers with a stronger Mn-C-H agostic interaction, but only a weak Mn-C-H agostic interaction could be presented in complexes **3** and **4**. Therefore, the order of the relative strengths of the Mn-C-H agostic interaction in complexes **1**, **2**, **3** and **4** is **1** ~ **2** > **4** > **3**.

The computed proton shifts showed that Hs in the Mn-H-C agostic unit of agostomers **1** (−9.8 ppm) and **2** (−9.9 ppm) were more shielded to those in the agostomers **3** (−5.5 ppm) and **4** (−8.7 ppm) (Table 1). Upon the introduction of extra magnetic fields, the agostomers **1** and **2** with the η^4^-(MeC_6_H_8_) fragment could form the pseudo ring current, generating outside-the-ring deshielded protons (non-agostic protons) and inside-the-ring shielded protons (agostic protons) (Figure S3). A similar effect could also be proposed for agostomers **3** and **4**; however, the presence of the η^3^-(MeC_6_H_8_) fragment in agostomers **3** and **4** instead of the η^4^-(MeC_6_H_8_) fragment in the agostomers **1** and **2** made the inside-the-ring protons (agostic protons) less shielded. It is also notable that the Mn-H distances in agostomers **1** and **2** are 1.849 Å and 1.844 Å (Table 1), respectively, which suggests that the agostic H in the Mn-H-C agostic unit could be treated as a classical hydride. The shielding from the *d* orbitals of Mn to the agostic hydride is critical and nonnegligible, and the shielded agostic hydride at very upfield conditions is expected. However, the Mn-H distances in agostomers **3** and **4** are 2.051 Å and 1.958 Å, respectively, which is significantly longer that those in agostomers **1** and **2**. The relatively weak Mn-H interactions in agostomers **3** and **4** suggest that it should be an agostic proton in the Mn-H-C agostic unit instead of the hydride. Therefore, the more shielded agostic H in the Mn-H-C agostic unit in agostomers **1** and **2** was observed.

### 2.2. Exchange Processes

Previous studies suggested that complexes **1** and **2** also were fluxional molecules [1]. However, possible multiple exchange processes made it difficult to distinguish each resonance in the variable temperature ^1^H NMR spectra. Only two exchange processes of the monomethyl cyclohexenyl manganese tricarbonyl (MeC_6_H_8_)Mn(CO)_3_) could be found in the experimentally obtained ^1^H NMR spectra: (1) a low-energy process estimated as 8.3 kcal/mol proceeding through the fast endo C-H exchange and (2) a high-energy exchange process estimated as 16.0 kcal/mol proceeding through the diene hydride species [1]. These values are quite similar to the parent system cyclohexenyl manganese tricarbonyl (C_6_H_9_)Mn(CO)_3_ (8.3 kcal/mol and 15.4 kcal/mol) [2]. In order to comprehensively interpret the ^1^H NMR spectra, explorations of the mechanisms in the exchange processes of complexes **1** and **2** were carried out. The following possible exchange processes were examined: agostomers conformational isomerization, conversion between agostomers and hydride species, and hydride species conversions.

#### 2.2.1. Isomerization of η^3^ Agostomers

**Figure 3:**
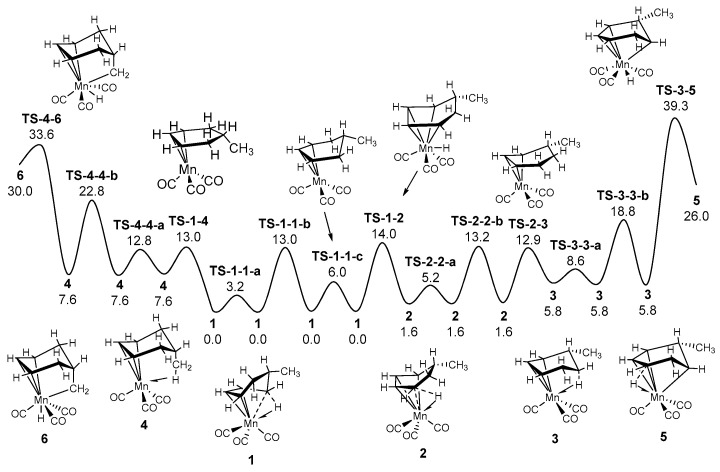
The free energy diagram of η^3^ agostomers isomerization of (MeC_6_H_8_)Mn(CO)_3_.

The first exchange processes explored were the self-isomerization of 6-monomethyl agostomer (complex **1**) and the rotation transition states (**TS-1-1-a**, methyl rotation, and **TS-1-1-b**, CO’s rotation, Figure 3). As expected, the magnitude of the rotational barrier for the methyl group (**TS-1-1-a**, 3.2 kcal/mol, all following energies were relative to complex **1**) was much less than that of the CO’s rotation (**TS-1-1-b**, 13.0 kcal/mol). Similar rotation transition states (methyl rotation, **TS-2-2-a**, 5.2 kcal/mol, and **TS-2-2-b**, CO’s rotation, 13.2 kcal/mol) for 5-monomethyl agostomer (complex **2**) were also observed. The conversion between complex **1** and complex **2** was accomplished by an η^4^-diene hydride transition state (**TS-1-2**, 14.0 kcal/mol), which is consistent with the experimentally proposed high-energy exchange process (16.0 kcal/mol) [1]. Another self-isomerization of 6-monomethyl agostomer (complex **1**) involved a *C_s_* symmetrical η^3^-allyl transition state (**TS-1-1-c**, 6.0 kcal/mol). This low activation energy η^3^-allyl transition state caused the fast exchange of the two endo H’s adjacent to the terminal positions of the allylic unit. The experimentally estimated Gibbs barrier for this fast endo C-H exchange process was 8.3 kcal/mol [1]. Since the endo methyl exists in the 5-monomethyl agostomers (complex **2**), the endo H’s exchange via the similar η^3^-allyl half-chair transition state was unachievable. However, the neighboring endo H’s exchange (1H_endo_ and 6H_endo_) of complex **2** was achieved by the *C_1_* symmetrical η^3^-allyl transition state (**TS-2-3**, 12.9 kcal/mol), and it generated another η^3^ complex **3**. Another endo H exchange between 5H_endo_ and 7-methyl_endo_, **TS-1-4** was also found, which produced another η^3^ complex **4**. The bonding characters of the η^3^ complexes **3** and **4** are summarized in Table 1. Based on the criteria for the agostic bond [9,10,14], complexes **3** and **4** could also be assigned as other agostomers with weaker agostic interaction compared to complexes **1** and **2**. The computed relative Gibbs free energies for the methyl and CO’s rotation of complex **3** (**TS-3-3-a**, methyl rotation, and **TS-3-3-b**, CO’s rotation) and complex **4** (**TS-4-4-a**, methyl rotation, and **TS-4-4-b**, CO’s rotation) are shown in Figure 3. Notably, the Gibbs barriers for the CO’s rotation in complexes **3** (**TS-3-3-b**, 18.8 kcal/mol) and **4** (**TS-4-4-b**, 22.8 kcal/mol) are significantly higher than those in complexes **1** (**TS-1-1-b**, 13.0 kcal/mol) and **2** (**TS-2-2-b**, 13.2 kcal/mol), which are caused by the steric hindrance in agostomers **3** and **4** with the “closed (MeC_6_H_8_)Mn fragment” compared to the “open (MeC_6_H_8_)Mn fragment” in agostomers **1** and **2**. Similarly, the limited rotation of methyl group in the agostomer **3** (**TS-3-3-a**, 8.8 kcal/mol) and agostomer **4** (**TS-4-4-a**, 12.8 kcal/mol) compared to those in agostomers **1** (**TS-1-1-a**, 3.2 kcal/mol) and **2** (**TS-2-2-a**, 5.2 kcal/mol) is also observed (Figure 3). The unfavorable endo-H migration from the methyl group in complex **4** to the Mn center (**TS-4-6**, 33.6 kcal/mol) forms the methylene hydride complex **6** (30.0 kcal/mol). The endo-H migration from the Mn-H-C unit in complex **3** to the Mn center (**TS-3-5**, 39.3 kcal/mol) forms another η^4^ agostomer **5** (26.0 kcal/mol). Since the C-H bond breaking is required for the H migration (**TS-4-6**, and **TS-3-5**), the relatively high Gibbs barriers are anticipated and observed. The participation of agostomer **5** and hydride complex **6** in the experimentally observed exchange process is excluded due to the high Gibbs barriers (39.3 kcal/mol for **TS-3-5** and 33.6 kcal/mol for **TS-4-6**, Figure 3).

For comparisons, additional single point energy calculations with lager basis sets (BS2 and BS3) were performed (Figure S1). The computational results showed that the mean signed deviation (MSD) and the mean absolute deviation (MAD) between PBEPBE/BS2-Auto//PBEPBE/BS1-Auto computations and PBEPBE/BS1-Auto computations are 0.3 and 0.5 (MSD = 0.3, MAD = 0.5), respectively. The MSD and MAD between PBEPBE/BS3-Auto//PBEPBE/BS1-Auto computations and PBEPBE-D3(BJ)/BS1-Auto computations are 0.2 and 0.5 (MSD = 0.2, MAD = 0.5), respectively. The excellent linear fitting of the computed Gibbs energies (Figure S2) was also obtained. The relatively small MSD and MAD values demonstrated the reliability of the DFT method utilized in this study (PBEPBE/BS1-Auto).

#### 2.2.2. Hydride Species Conversions

**Figure 4:**
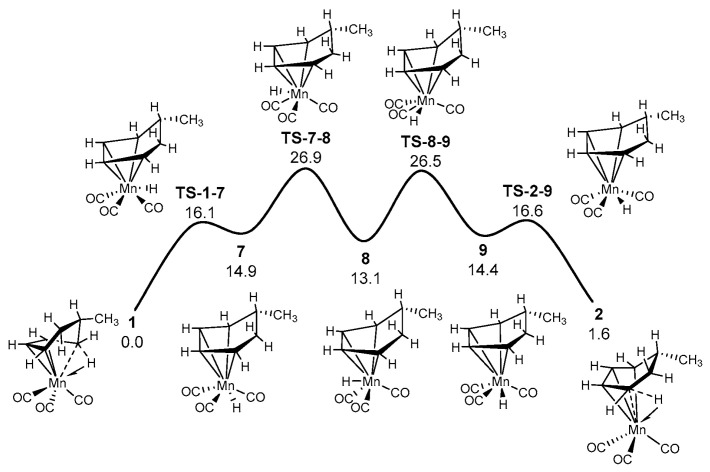
The second pathway of η^3^ agostomers isomerization of (MeC_6_H_8_)Mn(CO)_3_.

Another proposed pathway for the conversion between complex **1** and complex **2** was accomplished via three different η^4^-diene hydride minima (complexes **7**, **8**, and **9**, Figure 4). It was noted that computed energies of these three η^4^-diene hydride minima (14.9 kcal/mol for **7**, 13.1 kcal/mol for **8**, and 14.4 kcal/mol for **9**) were close to that of **TS-1-2** (14.0 kcal/mol) due to the structural similarity. However, the relatively high Gibbs barriers for **TS-7-8** (26.9 kcal/mol) and **TS-8-9** (26.5 kcal/mol) prevent the practical conversion between complex **1** and complex **2** (Figure 4).

### 2.3. Interpretations of the Fluxionality

**Scheme 3:**
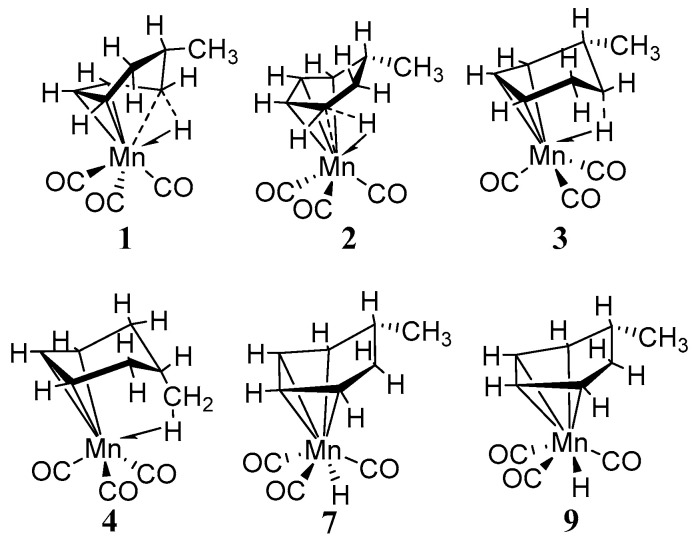
Structures involved in the fluxional processes of (MeC_6_H_8_)Mn(CO)_3_.

Based on the computational results, several important facts about the fluxionalities of the monomethyl cyclohexenylmanganese tricarbonyl [(MeC_6_H_8_)Mn(CO)_3_, complex **1** and **2**] were discovered. First, the methyl rotational energy barriers in complexes **1**, **2**, **3**, and **4** showed different patterns. Compared to complexes **1** (3.2 kcal/mol, energy relative to complex **1**), **2** (3.6 kcal/mol, relative to complex **2**), and **3** (2.8 kcal/mol, relative to complex **3**), complex **4** had much higher methyl rotational activation energy (5.3 kcal/mol, relative to complex **4**). This fact supported the conclusion on the weak M-H-C agostic interaction in complex **4**. Unlike the free methyl group in complexes **1**, **2**, and **3**, the weak Mn-H-C agostic interaction in complex **4** made two sets of inequivalent H toms (1 endo H and 2 exo H’s). The next fluxional process was the rotation of Mn(CO)_3_ fragment. The higher reaction free energies of the weak Mn-H-C agostomer **4** compared to agostomer **1**, **2**, and **3** made it exclude from this CO’s ligand equivalence process. Although the 5-monomethyl agostomer (complex **2**) could not adopt the similar η^3^-allyl transition state (**TS-3**) of 6-monomethyl agostomer to perform the fast exchange of the two endo Hs adjacent to the terminal positions of the allylic unit, the neighboring endo H exchange (1H_endo_ and 6H_endo_) of complex **2** was achieved by a higher energy *C*_1_ symmetrical η^3^-allyl transition state (**TS-2-3**, 12.9 kcal/mol). Another endo H exchange in complex **1** was the exchange between 5H_endo_ and methyl_endo_, **TS-1-4**. The conversion between complex **1** and complex **2** could be accomplished by a single H transfer process (**TS-1-2**, 14.0 kcal/mol) or by a series of three η^4^-diene hydride minima. However, the relative high activation energy of the second pathway suggested the high temperature conversion between complex **1** and complex **2** could only occur through η^4^-diene hydride transition state, **TS-1-2**. Notably, the conversion between complexes **1**, **2** and the η^4^-diene hydride minima, complexes **7** and **9** (Scheme 3) could complicate the ^1^H NMR spectrum of (MeC_6_H_8_)Mn(CO)_3_ in high temperature conditions. In other words, the fluxionalities of the monomethyl cyclohexenyl manganese tricarbonyl [(MeC_6_H_8_)Mn(CO)_3_] contained multiple exchange processes: (1) methyl rotation (**TS-1-1-a**, **TS-2-2-a**, **TS-3-3-a**, and **TS-4-4-a**); (2) CO’s ligand equivalence (**TS-1-1-b** and **TS-2-2-b**); (3) fast exchange of the endo Hs adjacent to the terminal positions of the allylic unit in complex **1** (**TS-3**); (4) low exchange of the neighboring endo H’s in complex **2** (**TS-2-3**); (5) low endo H exchange between 5H_endo_ and methyl_endo_ in complex **1** (**TS-1-4**); (6) 1, 2-agostic isomerization (**TS-1-2**); and (7) possible high temperature H atom migration of the agostic Mn-H-C unit (**TS-1-7** and **TS-2-9**).

**Figure 5:**
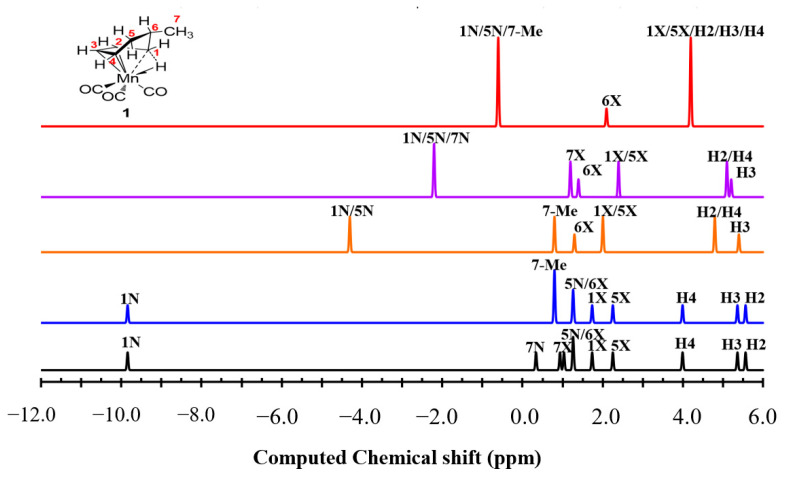
Simulated gas phase ^1^H NMR spectra of (MeC_6_H_8_)Mn(CO)_3_ at low temperature limit (black), low temperature exchange (blue), medium temperature exchange (orange), high temperature fast exchange (purple), and highest temperature fast exchange (red).

**Figure 6:**
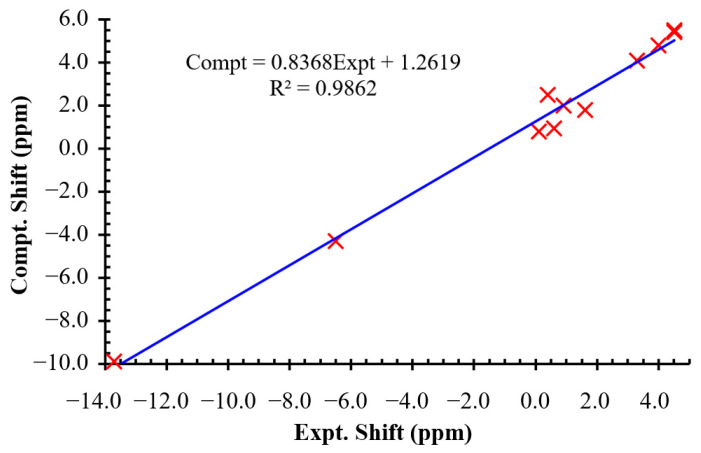
Linear fitting between the computed proton chemical shifts and experimental ^1^H NMR [1] of complexes **1** and **2**.

Based on the discussion above, the gas phase variable temperature ^1^H NMR spectra of (6-MeC_6_H_8_)Mn(CO)_3_ and (5-MeC_6_H_8_)Mn(CO)_3_ were simulated (Figure 5). An excellent linear relationship (R^2^ = 0.9862) between the computed proton chemical shifts and the experimental ^1^H NMR chemical shifts (Figure 6) was reached. The simulated low temperature exchange (methyl rotation **TS-1-1-a**) and medium temperature exchange (fast exchange of the endo H’s adjacent to the terminal positions of the allylic unit, **TS-1-1-c**) showed that the resonance of the methyl group was 0.77 ppm, which was close to the experimental reported methyl group (0.6 ppm) of complex **2**, 5-methyl (0.8 ppm) and 6-methyl (0.7 ppm) of the 5, 6-dimethyl analogue. In medium temperature, the *C_s_* symmetrical **TS-3** made 2H and 3H equivalent, 1H_endo_ and 4H_endo_ equivalent, and 1H_exo_ and 4H_exo_ equivalent, which gave six peaks in the ^1^H NMR spectrum. In the next high temperature regime, slow endo H exchange between the H5_endo_ and methyl_endo_ (**TS-1-4**) process averaged the 1H_endo_, 5H_endo_ and methyl_endo_. Unlike the two resonances in the super-high temperature ^1^H NMR of the unsubstituted analogue [(C_6_H_9_)Mn(CO)_3_] [5], the asymmetric 1, 2-agostic isomerization (**TS-1-2**) process finally gave three peaks for the highest temperature in the ^1^H NMR spectrum.

## 3. Computational Methods

Molecular structures were optimized in the gas phase using the Perdew, Burke, and Ernzerhof exchange functional and gradient-corrected correlation functional (PBEPBE) [15] with basis set 1 and the density fitting approximation [16,17] (BS1, the modified-LANL2DZ with the *f* polarization [(modified-LANL2DZ(f)] [18,19,20] and related effective core potential (ECP, LANL2DZ) for Mn atom, LANL2DZ(*d*, *p*) [21,22] with the related LANL2DZ ECP for Si atom of the reference TMS, and the 6-31G (*d*′) [23,24,25] for all other atoms (C, O, and H)). All computations (PBEPBE/BS1-Auto) were carried out with Gaussian 09 software (Revision C01) [26]. Pruned fine integration grids with 75 radial shells and 302 angular points per shell were used for all computations. Free energy corrections were computed at 1 atm and 298.15 K. Optimization using Grimme’s D3 [27] dispersion with Becke–Johnson damping (D3(BJ)) [28] was also compared (PBEPBE-D3(BJ)/BS1-Auto). For comparisons, the single-point energy calculations using Def2-TZVPP basis sets (BS3, Def2-TZVPP for Mn, C, O, and H) for serval salient transition states were also performed (see Supplementary Materials, Figures S1 and S2). All located minima were verified by vibrational frequency computations with no imaginary frequency, and all located transition states were obtained with only one imaginary frequency. The intrinsic reaction coordinate computations from the located transition states were performed, and both directions of the reaction path following the transition state were computed [29]. The electron density of the bond critical point [ρ_(BCP)_] based on Bader’s theory of atoms-in-molecules (AIM) [12,30,31] was calculated with the Multiwfn package (version 3.8) [32,33], and were visualized with the VMD package (version 1.9.3) [34,35]. Notably, the reliability of quantum mechanics (QM) computation instead of molecular dynamics (MD) simulation in the study of the fluxional processes of organometallics has been established [5,36].

The gauge-independent atomic orbital (GIAO) [37,38,39] method with the PBEPBE functional and basis set 2 (BS2, LANL08(*f*) [20,40] basis set and the related LANL2DZ ECP for Mn, LANL08(*d*) [22,40] and related LANL2DZ ECP for Si, and the 6-311G++(3*df*, 3*pd*) [41,42] basis sets for C, O, and H) was used to compute the magnetic shielding tensors of the gas-phase optimized structures.

## 4. Conclusions

Compared to the unsubstituted analogue [(C_6_H_9_)Mn(CO)_3_], multiple exchange processes in the fluxionalities of the monomethyl cyclohexenyl manganese tricarbonyl [(MeC_6_H_8_)Mn(CO)_3_] were revealed. First, two different conversion pathways between agostomer **1** (6-monomethyl) and agostomer **2** (6-monomethyl) were located: (1) via the η^4^-diene hydride transition state; and (2) via three η^4^-diene hydride minima. The computational results demonstrated that the 1, 2-agostic isomerization only occurred via the η^4^-diene hydride transition state (**TS-1-2**, 14.0 kcal/mol). The previously proposed two exchange processes, including a low-energy fast endo C-H exchange and a high-energy exchange process proceeding through the diene hydride species, were verified. The computed Gibbs barriers are 6.0 kcal/mol and 14.0 kcal/mol, respectively, for these exchange processes, which are consistent with the experimentally estimated barriers (8.3 kcal/mol and 16.0 kcal/mol). Other exchange processes, such as methyl group rotation, CO ligand equivalence, fast exchange of the endo H’s in agostomer **1**, low exchange of the neighboring endo H in agostomer **2**, low endo H exchange between 5H_(endo)_ and methyl_(endo)_ in complex **1**, and the possible high temperature H atom migration of the agostic Mn-H-C unit were also studied. Moreover, the bonding characters and the AIM (atoms-in-molecules) analyses of complexes **3** and **4** demonstrated **3** and **4** also were agostomers with weak Mn-H-C agostic interaction compared to agostomers **1** and **2**. Finally, the gas-phase variable temperature ^1^H NMR spectra of (MeC_6_H_8_)Mn(CO)_3_ based on the exchange processes provided were simulated, and the detailed resonances were revealed. These results could potentially establish fundamental insights into the role of agostic interaction in the homogeneous catalysis, especially with regard to transition metal catalyzed C-H activation.

## Figures and Tables

**Table 1:** Comparisons of agostic parameters of complex **1**, **2**, **3**, and **4**. The experimentally derived Mn-H and Mn-H-C parameters of complex 1 (CSD entry: BEZYEK) [2] are given in parentheses.

Structures	Mn-H (Å)	Mn-H-C (°)	C-H (Å)		H (ppm)		J_CH_ (Hz)	
			endo	exo	endo	exo	endo	exo
1	1.849 (1.860)	97.6 (99.9)	1.180	1.104	−9.8	1.7	68.4	123.3
2	1.844	97.9	1.179	1.103	−9.9	1.8	68.8	124.6
3	2.051	104.6	1.136	1.107	−5.2	0.4	83.1	117.6
4	1.958	130.0	1.131	1.105	−8.7	1.5	82.3	113.5

## Data Availability

Not applicable.

## Supplementary Materials

The following supporting information can be downloaded at: https://www.mdpi.com/article/10.3390/molecules28073232/s1, Table S1: the matched DFT optimized structure; Table S2: Selected bond lengths and angles; Table S3: DFT optimized structures; Table S4: Simulated chemical shifts of complex **1**; Table S5: Simulated chemical shifts of complex **2**; Table S6: DFT computed gas phase energies; Table S7: DFT single-point energies; Table S8: Cartesian coordinates of optimized structures; Figure S1: The free energy diagram; Figure S2: Linear relationship of Gibbs energies; Figure S3: Illustration of the shielded and deshielded protons; Figure S4: IRC plots for **TS-3**; Figure S5: IRC plots for **TS-2-3**; Figure S6: IRC plots for **TS-1-4**; Figure S7: Computed Wiberg bond index; Figure S8: Computed proton chemical shifts; Figure S9: Simulated gas phase chemical shifts of complex **2**.

## Sample Availability

Samples of the compounds are not available from the authors.
