# Supplementary material for: Insights into the Fluxional Processes of Monomethylcyclohexenyl Manganese Tricarbonyl
Source: Molecules. 2023 Apr 4;28(7):3232. doi: 10.3390/molecules28073232 (PMC10096699; doi:10.3390/molecules28073232)
Supplement: Supplementary file 1 [file molecules-28-03232-s001.zip › molecules-2302211-supplementary.pdf]

--Supporting Information--

Insights into the Fluxional Processes of Monomethylcyclohexenylmanganese Tricarbonyl

Guangchao Liang[†]* and *Min Zhang*[‡]

[†]Academy of Advanced Interdisciplinary Research, Xidian University, Xi'an, Shaanxi 710071, P.R. China

[‡] Department of Pharmacy, School of Medicine, Xi'an International University, Xi'an, Shaanxi 710077, P.R. China

Table of Contents

Table S1. The matched DFT optimized structure with the reported X-ray crystal structure of (6-MeC_6H_8)$Mn(CO)_3$ S2

Figure S1. The free energy diagram of η^3 agostomers isomerization of (MeC_6H_8)$Mn(CO)_3$...... S2

Figure S2. Linear relationship of Gibbs energies between the PBEPBE/BS1-Auto computations and other computations. S3

Figure S3. Illustration of the shielded and deshielded protons upon extra magnetic field. S3

Table S2. Selected bond lengths and angles of (6-MeC_6H_8)$Mn(CO)_3$ and (5-MeC_6H_8)$Mn(CO)_3$ S4

Table S3. DFT optimized structures of (MeC_6H_8)$Mn(CO)_3$ species S5

Figure S4. IRC plots for TS-3. .... S6

Figure S5. IRC plots for TS-2-3. .... S6

Figure S6. IRC plots for TS-1-4. .... S7

Figure S7. Computed Wiberg bond index of complexes 1, 2, 3 and 4 .... S7

Figure S8. Computed proton chemical shifts (in ppm) of complexes 1, 2, 3, 4, 7, and 9 .... S8

Table S4. Simulated chemical shifts of complex 1 at variable temperatures S8

Table S5. Simulated chemical shifts of complex 2 at variable temperatures S8

Figure S9. Simulated gas phase chemical shifts of complex 2 at variable temperatures S9

Table S6. DFT computed gas-phase energies (in Hartrees) of (MeC_6H_8)$Mn(CO)_3$ species S9

Table S7. DFT single-point energies (in Hartrees) of (MeC_6H_8)$Mn(CO)_3$ species S9

Table S8. Cartesian coordinates of optimized structures of (MeC_6H_8)$Mn(CO)_3$ species S10

Table S1. The matched DFT optimized structure with the reported X-ray crystal structure of (6-MeC_6H_8)$Mn(CO)_3$.

Color code: yellow, Mn; red, O; gray, C; white, H. All non-hydrogen atoms are used to calculate the RMSD (in Å), X-ray crystal structures are presented in green.

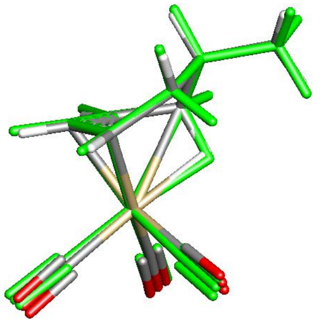 **RMSD = 0.037**<br>CSD entry: BEZYEK[ref]<br>PBEPBE/BS1-Auto Optimized	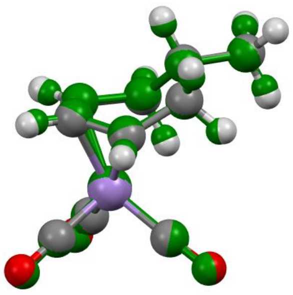 **RMSD = 0.034**<br>CSD entry: BEZYEK[ref]<br>PBEPBE-D3(BJ)/BS1-Auto Optimized

[ref] *J. Am. Chem. Soc.* **1982**, *104*, 2117-2126.

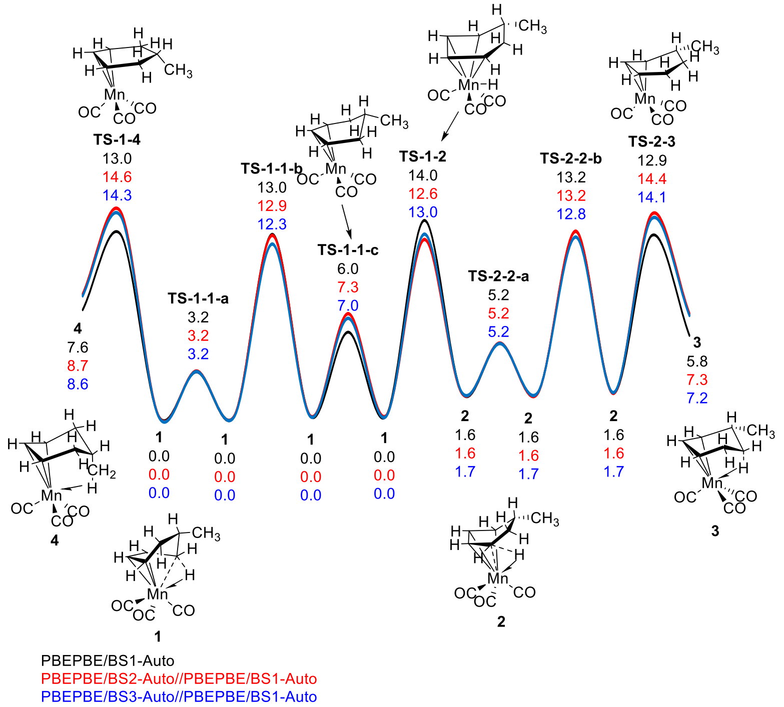

Figure S1. The free energy diagram of η^3 agostomers isomerization of (MeC_6H_8)$Mn(CO)_3$. $\Delta G°/\Delta G^‡$ are in kcal mol^{-1}. The black values are from PBEPBE/BS1-Auto computations, the red values are from PBEPBE/BS2-Auto//PBEPBE/BS1-Auto computations, and the blue values are from PBEPBE/BS3-Auto//PBEPBE/BS1-Auto computations. In BS1, the modified-LANL2DZ(f) and LANL2DZ ECP were used for Mn atom, and the 6-31G (d') basis sets were used for C, O, and H. In BS2, the LANL08(f) and LANL2DZ ECP were used for Mn atom, and

the 6-311G++(3df, 3pd) basis sets were used for C, O, and H. In BS3, the Def2-TZVPP basis sets were used for Mn, C, O, and H.

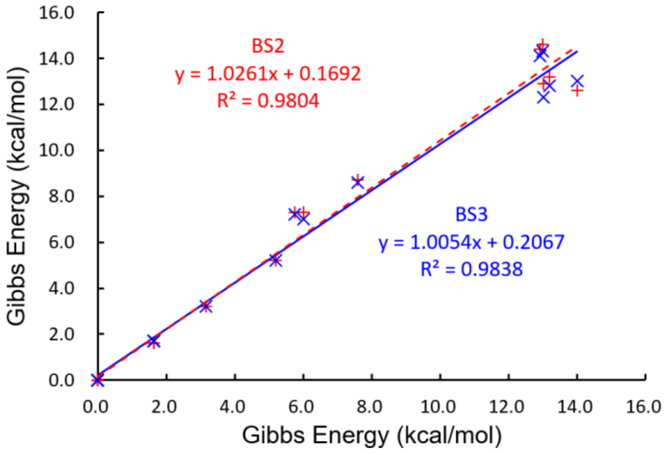

Figure S2. Linear relationship of Gibbs energies between the PBEPBE/BS1-Auto computations and other computations.

The red values are from PBEPBE/BS2-Auto//PBEPBE/BS1-Auto computations, and the blue values are from PBEPBE/BS3-Auto//PBEPBE/BS1-Auto computations. The computational results showed that the mean signed deviation (MSD) and the mean absolute deviation (MAD) between PBEPBE/BS2-Auto//PBEPBE/BS1-Auto computations and PBEPBE/BS1-Auto computations are 0.3 and 0.5 (MSD = 0.3, MAD = 0.5), respectively. The MSD and MAD between PBEPBE/BS3-Auto//PBEPBE/BS1-Auto computations and PBEPBE-D3(BJ)/BS1-Auto computations are 0.2 and 0.5 (MSD = 0.2, MAD = 0.5), respectively.

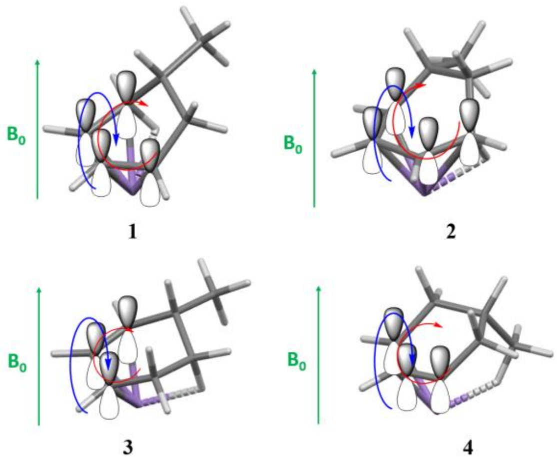

Figure S3. Illustration of the shielded and deshielded protons upon extra magnetic field.

Table S2. Selected bond lengths and angles of (6-MeC_6H_8)Mn$(CO)_3$ and (5-MeC_6H_8)Mn$(CO)_3$.

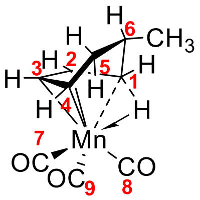

1

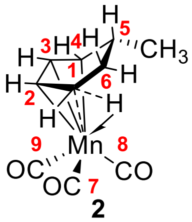

2

**Parameter**	**1**			**2**	
	Expt. [ref]	Compt.#	Compt.*	Compt.#	Compt.*
Mn-C1	2.301	2.321	2.310	2.322	2.316
Mn-C2	2.053	2.054	2.048	2.059	2.049
Mn-C3	2.092	2.094	2.089	2.092	2.087
Mn-C4	2.168	2.172	2.167	2.181	2.174
Mn-C7	1.775	1.761	1.783	1.792	1.789
Mn-C8	1.814	1.787	1.791	1.789	1.785
Mn-C9	1.804	1.794	1.758	1.761	1.757
Mn-H1(endo)	1.859	1.849	1.840	1.844	1.841
C1- H1(endo)	1.072	1.180	1.180	1.179	1.178
C1- H1(exo)	1.032	1.104	1.103	1.103	1.102
C1-C6	1.530	1.549	1.547	1.544	1.543
C1-C2	1.485	1.503	1.501	1.503	1.502
C3-C4	1.400	1.430	1.422	1.431	1.430
C4-C5	1.514	1.528	1.526	1.536	1.534
C1- H1(endo)-Mn	99.9	97.6	97.4	97.9	97.8
C2-C1-C6	114.6	113.8	113.9	112.8	112.7
C3-C4-C5	120.8	120.4	120.5	120.1	120.1

where Compt.# means the optimized structure from PBEPBE/BS1-Auto and Compt.* means the optimized structure from PBEPBE-D3(BJ)/BS1-Auto. Bond lengths are given in Å and bond angles are in °. [ref] *J. Am. Chem. Soc.* **1982**, *104*, 2117-2126.

Table S3. DFT optimized structures of $(MeC_6H_8)Mn(CO)_3$ species

**1**	**2**	**3**	**5**	**4**
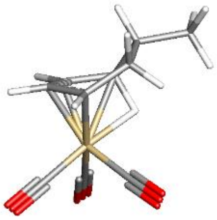	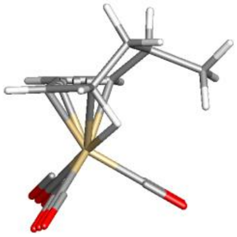	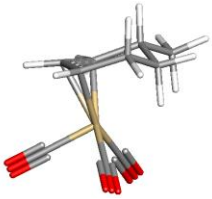	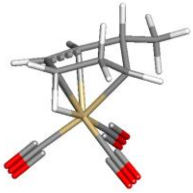	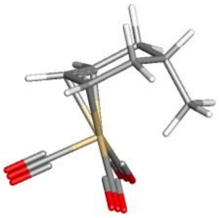
**6**	**7**	**8**	**9**	**TS-1-1-a**
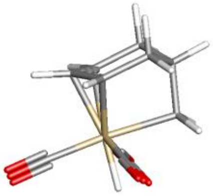	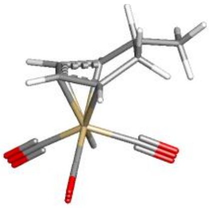	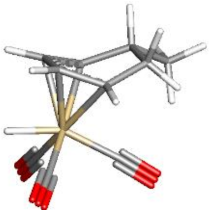	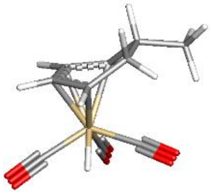	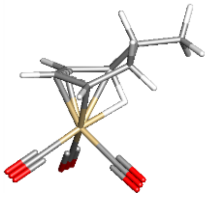
**TS-1-1-b**	**TS-1-1-c**	**TS-1-2**	**TS-2-2-a**	**TS-2-2-b**
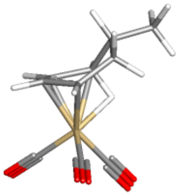	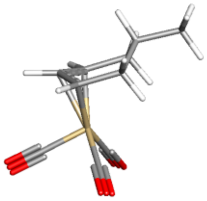	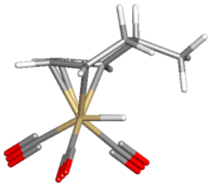	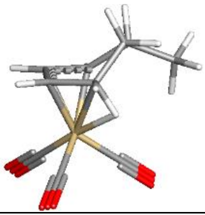	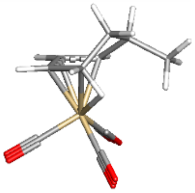
**TS-2-3**	**TS-3-3-a**	**TS-3-3-b**	**TS-3-5**	**TS-1-4**
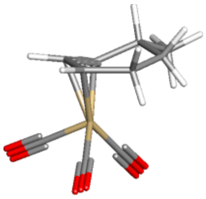	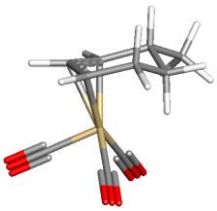	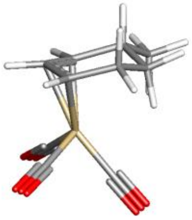	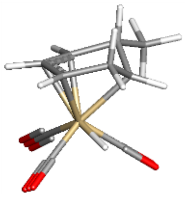	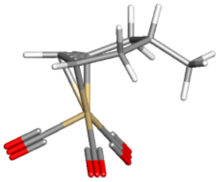
**TS-4-4-a**	**TS-4-4-b**	**TS-4-6**	**TS-1-7**	**TS-7-8**
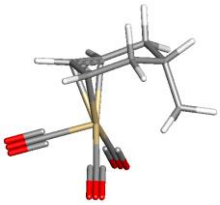	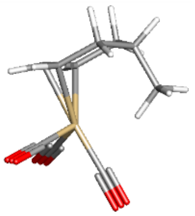	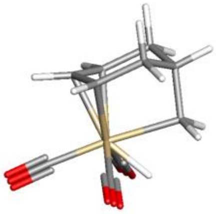	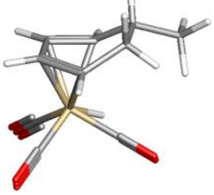	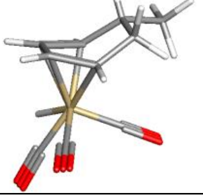
**TS-2-9**	**TS-8-9**			
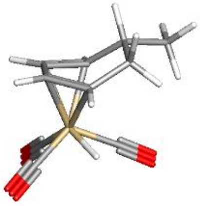	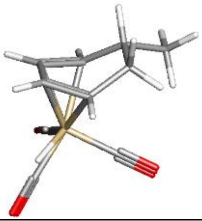			

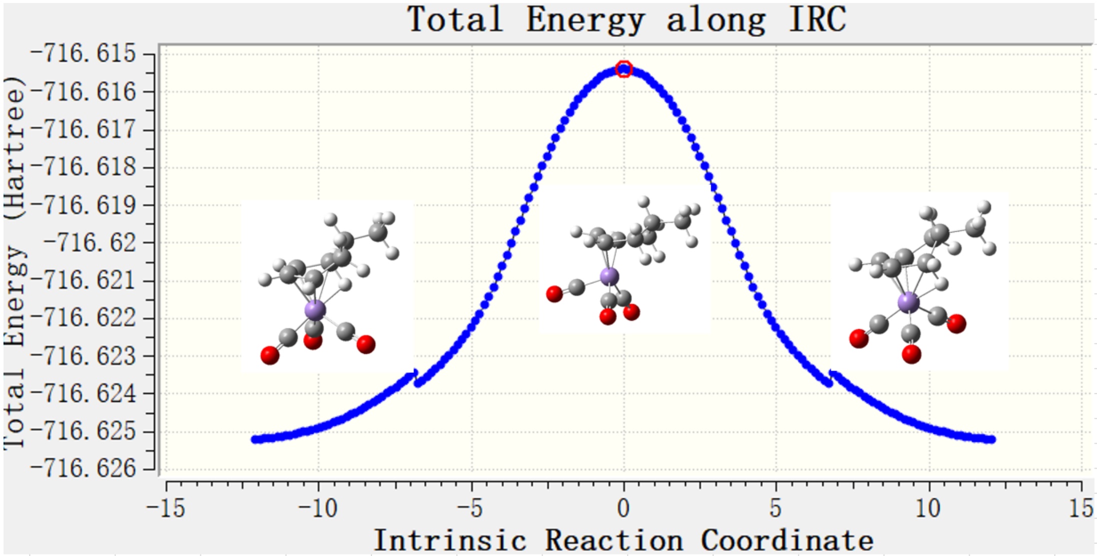

Figure S4. IRC plots for **TS-3**.

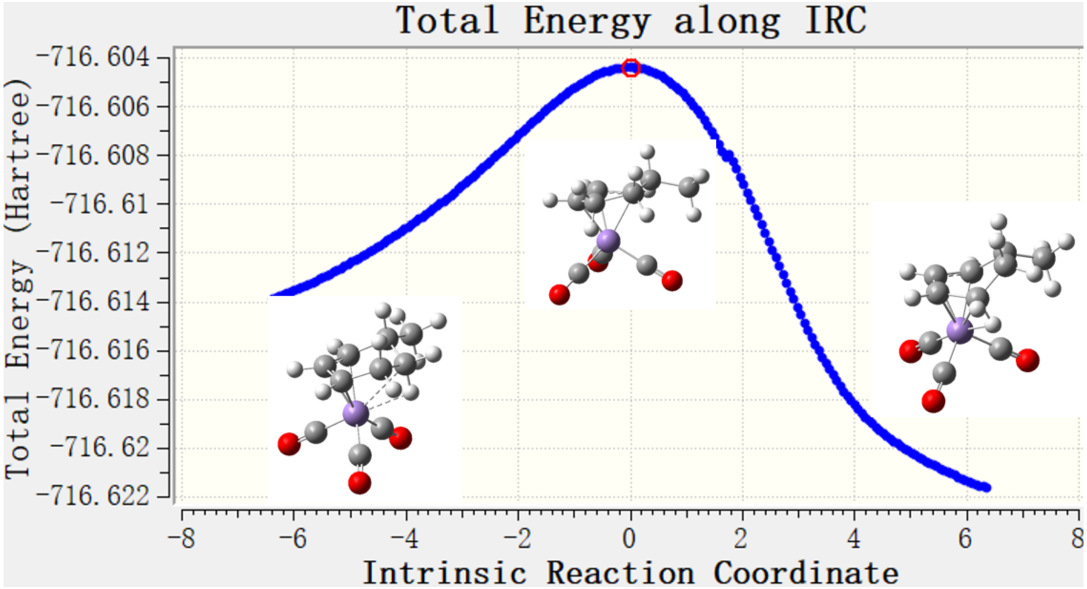

Figure S5. IRC plots for **TS-2-3**.

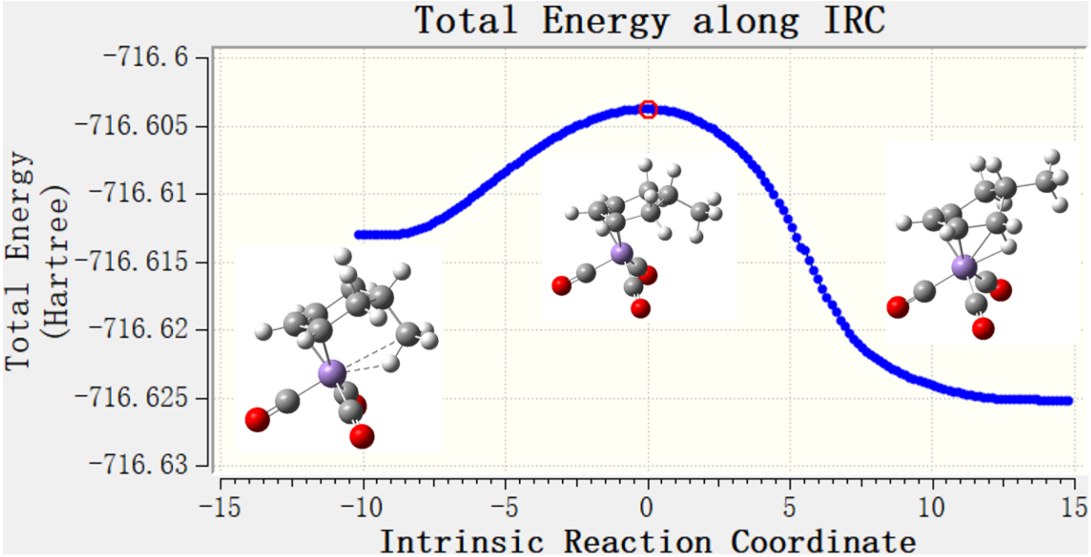

Figure S6. IRC plots for **TS-1-4**.

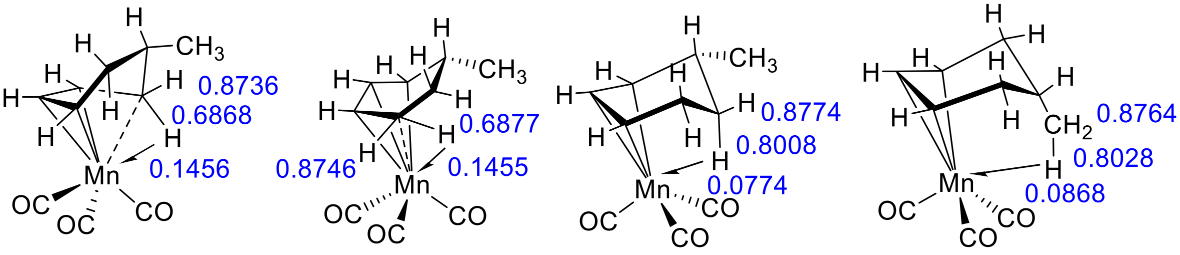

1 **2** **3** **4**

Figure S7. Computed Wiberg bond index of complexes 1, 2, 3 and 4.

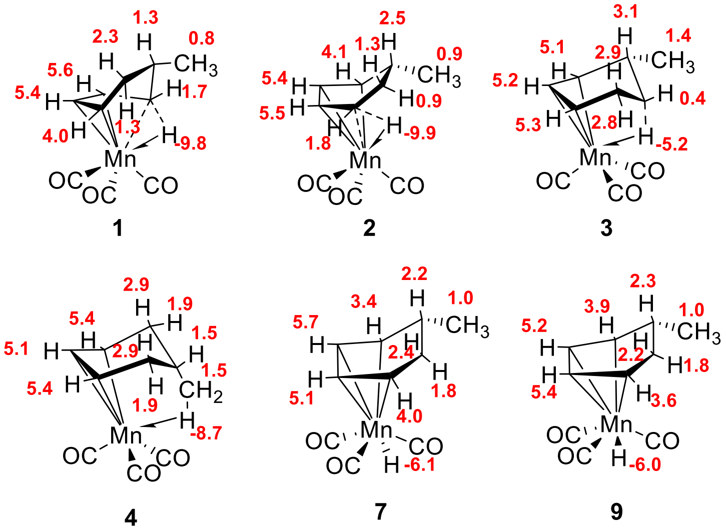

Figure S8. Computed proton chemical shifts (in ppm) of complexes 1, 2, 3, 4, 7, and 9.

Table S4. Simulated chemical shifts of complex 1 at variable temperatures.

Chemical shift (ppm)	1N	5N	7N	7X	1X	5X	H2	H4	H3	6X
Low temperature limit	-9.8	1.3	0.3	1.0	1.7	2.3	5.6	4.0	5.4	1.3
[a] Low temperature	-9.8	1.3	0.8		1.7	2.3	5.6	4.0	5.4	1.3
[b] Medium temperature	-6.5		0.8		0.9		4.8		5.4	1.3
[c] High temperature	-2.2			1.2	2.4		5.1		5.2	1.4
[d] Super-high temperature	-0.6				4.2					2.1

[a] TS-1-1-a involved. [b] TS-1-1-c involved. [c] TS-1-4 involved. [d] TS-1-2 involved.

Table S5. Simulated chemical shifts of complex 2 at variable temperatures.

Chemical shift (ppm)	1N	6N	1X	6X	H2	H3	H4	5X	Me
Low temperature limit	-9.9	0.9	1.8	1.3	5.5	5.4	4.1	2.5	1.2, 0.8, 0.8
Low temperature a	-9.9	0.9	1.8	1.3	5.5	5.4	4.1	2.5	0.9
High temperature b	-2.8		1.6		5.4	5.3	4.6	2.8	1.2

[a] TS-2-2-a involved. [b] TS-2-3 involved

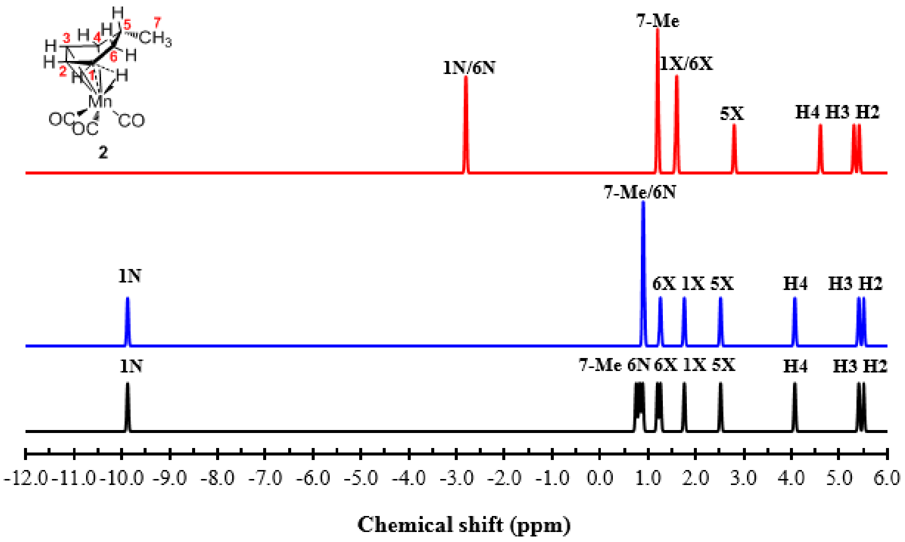

Figure S9. Simulated gas phase chemical shifts of complex 2 at variable temperatures. Low temperature limit (bottom, black), low temperature exchange (middle, blue), high temperature fast exchange (top, red).

Table S6. DFT computed gas-phase energies (in Hartrees) of $(MeC_6H_8)Mn(CO)_3$ species

Molecule	$\mathbf{E}_{(PBEPBE/BS1-Auto)}$	$\mathbf{E0}_{(PBEPBE/BS1-Auto)}$	$\mathbf{G}_{(PBEPBE/BS1-Auto)}$
Complex **1**	-716.6286332	-716.442661	-716.483694
Complex **2**	-716.6263688	-716.440250	-716.481082
Complex **3**	-716.6197652	-716.433628	-716.474520
Complex **5**	-716.5863525	-716.401570	-716.442196
Complex **4**	-716.6165576	-716.430560	-716.471666
Complex **6**	-716.5788365	-716.396026	-716.435859
Complex **7**	-716.6007338	-716.418732	-716.459902
Complex **8**	-716.6039344	-716.421359	-716.462806
Complex **9**	-716.6014283	-716.419423	-716.460734
**TS-1-1-a**	-716.6237453	-716.438059	-716.478674
**TS-1-1-b**	-716.6083749	-716.423079	-716.462964
**TS-1-1-c**	-716.6185452	-716.433237	-716.474088
**TS-1-2**	-716.6038630	-716.421531	-716.461388
**TS-2-2-a**	-716.6209085	-716.435043	-716.475376
**TS-2-2-b**	-716.6080853	-716.422800	-716.462597
**TS-2-3**	-716.6075688	-716.422046	-716.463128
**TS-3-3-a**	-716.6153531	-716.429520	-716.470039
**TS-3-3-b**	-716.5985036	-716.413121	-716.453822
**TS-3-5**	-716.5630893	-716.381798	-716.421015
**TS-1-4**	-716.6069690	-716.421283	-716.463061
**TS-4-4-a**	-716.6084685	-716.422559	-716.463288
**TS-4-4-b**	-716.5919148	-716.406771	-716.447334
**TS-4-6**	-716.5716748	-716.389854	-716.430092
**TS-2-9**	-716.5990093	-716.417322	-716.457206
**TS-8-9**	-716.5837582	-716.401116	-716.441464
**TS-7-8**	-716.5832039	-716.400506	-716.440795
**TS-1-7**	-716.5997033	-716.418102	-716.458078

Table S7. DFT single-point energies (in Hartrees) of $(MeC_6H_8)Mn(CO)_3$ species

Molecule	$\mathbf{E}_{(PBEPBE/BS3-Auto)}$	$\mathbf{E}_{(PBEPBE/BS3-Auto)}$	$\mathbf{G}_{Corr\ (PBEPBE/BS1-Auto)}$
Complex **1**	-716.8744282	-1763.75233	0.144939
Complex **2**	-716.8721596	-1763.750032	0.145287
Complex **3**	-716.8630838	-1763.741172	0.145245
Complex **4**	-716.8604957	-1763.738561	0.144891
**TS-1-1-a**	-716.8694648	-1763.747398	0.145072

**TS-1-1-b**	-716.8543373	-1763.733127	0.145411
**TS-1-1-c**	-716.8622429	-1763.740566	0.144332
**TS-1-2**	-716.8519587	-1763.729087	0.142475
**TS-1-4**	-716.8501161	-1763.728498	0.143908
**TS-2-3**	-716.8509584	-1763.729398	0.144440
**TS-2-2-a**	-716.866683	-1763.744599	0.145533
**TS-2-2-b**	-716.8538943	-1763.732499	0.145488

Table S8. Cartesian coordinates of optimized structures of $(MeC_6H_8)Mn(CO)_3$ species

```
25
Complex 1    el energy= -716.628633192
Mn          1.971779        2.268582        0.891367
O           3.177517        1.566186       -1.721031
O          -0.271583        3.431610       -0.593625
O           0.419281       -0.176210        1.476333
C           3.973541        2.372578        2.060506
C           3.397979        3.629251        1.470017
C           2.148169        4.020641        2.024594
C           1.507036        3.055285        2.861777
C           2.309365        2.288542        3.912372
C           3.819553        2.277199        3.598830
C           4.530254        1.046443        4.175097
C           2.714834        1.833965       -0.682484
C           0.619659        2.961708        0.000587
C           1.024192        0.788568        1.216161
H           3.409855        1.410688        1.675067
H           4.995242        2.175989        1.691774
H           3.986248        4.268640        0.806263
H           1.633871        4.919399        1.662603
H           0.451914        3.228704        3.107938
H           1.934642        1.248992        3.991350
H           2.136581        2.748600        4.907326
H           4.284160        3.190008        4.019799
H           5.620011        1.082229        3.992191
H           4.140093        0.114402        3.722752
H           4.374602        0.974146        5.266842

25
Complex 2    el energy= -716.626368836
Mn          2.071631        2.348457        0.891359
O           2.887364        0.900773       -1.522400
```

O	0.298541	3.986963	-0.821265
O	0.175421	0.177774	1.550517
C	3.616042	1.630952	2.253863
C	4.085618	2.653213	1.369808
C	3.355719	3.873045	1.394055
C	2.366680	3.976151	2.520976
C	2.931555	3.461253	3.862898
C	3.303799	1.965737	3.719618
C	2.250357	1.038739	4.347354
C	2.556231	1.472763	-0.557276
C	0.990414	3.344202	-0.133871
C	0.925551	1.035015	1.292540
H	4.003034	0.616402	2.089898
H	1.898777	4.973602	2.568924
H	4.828165	2.462241	0.585035
H	3.601213	4.734704	0.767341
H	1.423734	3.292740	2.336972
H	2.192154	3.603695	4.672424
H	3.817110	4.071092	4.117746
H	4.253516	1.794160	4.271101
H	2.481367	-0.023508	4.147823
H	1.241172	1.237810	3.945405
H	2.212890	1.180822	5.443183

25
Complex 3   el energy= -716.619765231

Mn	2.200006	2.241347	1.084916
O	2.969413	2.370757	-1.728681
O	-0.562060	2.962251	0.303171
O	2.025762	-0.700205	0.800247
C	4.067980	2.253791	2.179766
C	3.812641	3.504710	1.550987
C	2.619153	4.213623	1.857333
C	1.990774	4.045299	3.232456
C	2.102428	2.562238	3.645011
C	3.550280	2.020631	3.598420
C	3.633440	0.553805	4.041645
C	2.656166	2.316545	-0.602052
C	0.524056	2.669542	0.618079
C	2.094796	0.459256	0.929772
H	4.936038	1.669640	1.850078
H	2.492845	4.692641	3.983108
H	4.405737	3.801676	0.675873
H	2.387347	5.120934	1.287936
H	0.928562	4.349939	3.209929

H	1.450071	1.925975	2.967328
H	1.636046	2.381238	4.632412
H	4.167715	2.619170	4.305240
H	4.673833	0.184775	3.987293
H	3.013560	-0.101794	3.404225
H	3.290885	0.439013	5.086464

25

Complex 4 el energy= -716.616557829

Mn	-0.911054	-0.114876	-0.873341
O	0.098102	-1.059966	-3.493637
O	-2.991680	1.320176	-2.332271
O	-2.967010	-2.131928	-0.178757
C	1.809616	0.708391	0.109082
C	0.657500	1.366112	-0.645997
C	-0.522309	1.703397	0.073348
C	-0.946796	0.804850	1.090968
C	0.114183	0.115118	1.944665
C	1.346128	-0.352140	1.134850
C	1.044842	-1.676330	0.428767
C	-0.298623	-0.703057	-2.454741
C	-2.156243	0.745292	-1.746098
C	-2.145906	-1.349205	-0.456652
H	2.524302	0.246258	-0.598334
H	2.373076	1.512362	0.630022
H	0.937902	1.992821	-1.501389
H	-1.179459	2.511574	-0.272438
H	-1.909211	0.996693	1.580909
H	-0.320783	-0.749311	2.481643
H	0.432865	0.833358	2.730661
H	2.178232	-0.540441	1.842520
H	1.910639	-2.044536	-0.151243
H	0.205939	-1.627426	-0.328285
H	0.722806	-2.461114	1.137338

25

Complex 5 el energy= -716.619765704

Mn	-0.580297	0.128580	0.096246
O	-2.652125	0.889899	2.007517
O	-2.705493	-0.622866	-1.821736
O	-0.304719	2.993346	-0.597227
C	1.239097	-0.001553	1.263022
C	0.322695	-1.039699	1.591253
C	-0.125710	-1.927170	0.575321
C	0.789314	-2.226113	-0.602571

C	1.500456	-0.920136	-1.015880
C	2.257061	-0.239422	0.148522
C	2.976885	1.039780	-0.299070
C	-1.824564	0.586913	1.237024
C	-1.861397	-0.320809	-1.072913
C	-0.398222	1.859209	-0.330933
H	1.487841	0.739817	2.032360
H	1.536846	-3.005121	-0.339993
H	-0.207079	-1.010078	2.552560
H	-0.909487	-2.652673	0.821215
H	0.204592	-2.620790	-1.453408
H	0.742131	-0.188279	-1.439034
H	2.162684	-1.079460	-1.888399
H	3.032011	-0.947004	0.519752
H	3.498534	1.515750	0.551159
H	2.271696	1.781571	-0.714834
H	3.733246	0.814755	-1.073300

25

Complex 6   el energy= -716.579020972

Mn	-0.505224	-0.000120	-0.116811
O	-0.823290	2.660062	-1.361774
O	-2.972152	-0.000183	1.530661
O	-0.822400	-2.661036	-1.360526
C	2.214705	1.264121	0.333224
C	0.897492	1.210858	1.086846
C	0.490669	0.000394	1.721257
C	0.898049	-1.210071	1.087194
C	2.215290	-1.262952	0.333587
C	2.350587	0.000490	-0.531510
C	1.174379	0.000071	-1.502856
C	-0.704125	1.628410	-0.830979
C	-1.983567	-0.000292	0.910208
C	-0.703458	-1.628943	-0.830529
H	2.249455	2.164123	-0.309100
H	3.048217	1.360273	1.061961
H	0.521816	2.148799	1.516586
H	-0.155509	0.000370	2.606131
H	0.522810	-2.148058	1.517222
H	2.250450	-2.163123	-0.308477
H	3.048852	-1.358512	1.062349
H	3.331364	0.000638	-1.053826
H	1.179022	0.887890	-2.160347
H	-1.534679	-0.000395	-1.279248
H	1.179413	-0.887932	-2.160091

25
Complex 7 el energy= -716.600733807

Mn	-0.791348	-3.056972	0.210881
O	0.899706	-2.984243	-2.214625
O	-3.059403	-2.601614	-1.619221
O	-2.161766	-5.454123	1.278547
C	0.565944	-2.960243	1.970591
C	0.610839	-1.807066	1.138914
C	-0.642476	-1.122459	0.970378
C	-1.750894	-1.725388	1.638572
C	-1.618252	-2.299422	3.039627
C	-0.210686	-2.897863	3.285730
C	-0.254343	-4.240632	4.029765
C	0.244222	-2.971446	-1.248192
C	-2.154723	-2.795959	-0.907575
C	-1.636232	-4.481728	0.906138
H	-0.068374	-4.383567	-0.180812
H	1.445827	-3.618002	1.962295
H	1.501963	-1.511087	0.574050
H	-0.752257	-0.290482	0.266871
H	-2.749941	-1.354370	1.372701
H	-2.395413	-3.071481	3.195528
H	-1.835783	-1.498373	3.777534
H	0.355649	-2.191167	3.930987
H	0.764850	-4.623773	4.222576
H	-0.803328	-5.006717	3.455351
H	-0.758827	-4.125799	5.006715

25
Complex 8 el energy= -716.603934445

Mn	0.110962	0.083502	0.288454
O	1.062823	2.843324	-0.155738
O	-1.153024	0.880344	2.850509
O	2.389303	-1.056870	1.777721
C	-1.679578	0.471437	-0.985538
C	-0.737069	-0.431957	-1.545827
C	-0.477299	-1.615101	-0.762947
C	-1.192311	-1.695734	0.464000
C	-2.646887	-1.268841	0.566131
C	-2.960618	-0.054149	-0.345136
C	-3.763937	1.039843	0.374358
C	0.692537	1.756220	0.040584
C	-0.709458	0.564796	1.819181
C	1.481114	-0.599454	1.206686

H	1.360997	0.028374	-0.661756
H	-1.758183	1.461820	-1.455473
H	-0.161463	-0.208378	-2.449717
H	0.311013	-2.322994	-1.037372
H	-0.866358	-2.460321	1.182008
H	-2.891007	-1.031411	1.618475
H	-3.287709	-2.134495	0.296703
H	-3.588283	-0.408720	-1.192349
H	-4.724418	0.637291	0.745113
H	-3.993214	1.880080	-0.306942
H	-3.210784	1.444839	1.239642

25
Complex 9 el energy= -716.601428271

Mn	-0.659487	-0.154075	-0.045418
O	-1.919345	2.308504	0.976011
O	0.241862	1.237321	-2.499311
O	-3.293983	-1.445545	0.326505
C	1.056076	0.340186	1.214929
C	0.321130	-0.795506	1.667450
C	0.260763	-1.883614	0.726442
C	0.907623	-1.660882	-0.517317
C	2.249408	-0.950688	-0.595537
C	2.384801	0.151121	0.486337
C	2.951004	1.465182	-0.070967
C	-1.440987	1.329267	0.558172
C	-0.063021	0.719175	-1.499245
C	-2.238861	-0.956476	0.220947
H	-1.403162	-0.582661	-1.350086
H	0.982279	1.252733	1.822856
H	-0.249268	-0.812084	2.601945
H	-0.366944	-2.762381	0.910989
H	0.689516	-2.369456	-1.327328
H	2.380036	-0.515294	-1.603988
H	3.057487	-1.704373	-0.486205
H	3.098458	-0.205614	1.261216
H	3.943612	1.298506	-0.527908
H	3.071207	2.219802	0.728295
H	2.289759	1.892615	-0.845145

25
TS-1-1-a el energy= -716.623745265

Mn	1.933366	2.261780	0.900326
O	3.063559	1.470442	-1.720175
O	-0.303349	3.466434	-0.560896

O	0.317613	-0.120736	1.565959
C	3.964016	2.325402	2.028670
C	3.412817	3.585893	1.421776
C	2.189345	4.028221	1.996526
C	1.537774	3.100028	2.866224
C	2.338130	2.318403	3.907197
C	3.844279	2.274759	3.574885
C	4.574098	1.058553	4.197585
C	2.630044	1.772481	-0.678436
C	0.585441	2.980003	0.023684
C	0.946241	0.819338	1.273582
H	3.363845	1.373737	1.679004
H	4.971180	2.092165	1.642414
H	4.007233	4.194397	0.734754
H	1.694932	4.935845	1.629089
H	0.494755	3.311238	3.133578
H	1.944995	1.286062	3.986703
H	2.184450	2.778402	4.905408
H	4.312448	3.206665	3.944271
H	5.344136	1.370390	4.924305
H	5.084036	0.446924	3.429758
H	3.873408	0.389319	4.728217

25

TS-1-1-b el energy= -716.608374910

Mn	1.882844	2.390913	0.885554
O	2.350021	0.111569	-0.919825
O	1.687434	4.136698	-1.464685
O	-0.966567	1.697701	0.965574
C	3.953587	2.476704	2.048287
C	3.233672	3.752906	1.724858
C	1.987704	3.926941	2.390676
C	1.470289	2.767414	3.039602
C	2.386046	1.868184	3.870867
C	3.874357	2.096173	3.550515
C	4.742407	0.877985	3.885845
C	2.154268	1.021978	-0.208349
C	1.779717	3.426728	-0.539595
C	0.178795	1.939496	0.933287
H	3.442880	1.555030	1.544504
H	4.968847	2.456524	1.616656
H	3.735522	4.564261	1.189536
H	1.377483	4.821026	2.217291
H	0.426725	2.807058	3.375893
H	2.121747	0.804771	3.712386

H	2.195646	2.073463	4.944985
H	4.238145	2.970205	4.125363
H	5.813514	1.078173	3.700281
H	4.450077	0.001454	3.276158
H	4.632773	0.594102	4.948307

25
TS-1-1-c el energy= -716.618545176

Mn	1.630699	-0.001354	0.000000
O	2.745954	1.537756	-2.272865
O	4.046693	-1.626830	0.000000
O	2.745954	1.537756	2.272865
C	-0.874988	0.076461	-1.247557
C	0.216814	-0.998814	-1.215350
C	0.441056	-1.706904	0.000000
C	0.216814	-0.998814	1.215350
C	-0.874988	0.076461	1.247557
C	-1.778602	0.030547	0.000000
C	-2.809855	1.166046	0.000000
C	2.301943	0.942751	-1.371230
C	3.081325	-0.962582	0.000000
C	2.301943	0.942751	1.371230
H	-0.442931	1.104492	-1.315848
H	-1.465837	-0.036428	-2.177667
H	0.493803	-1.469666	-2.164573
H	0.979708	-2.662435	0.000000
H	0.493803	-1.469666	2.164573
H	-0.442931	1.104492	1.315848
H	-1.465837	-0.036428	2.177667
H	-2.308393	-0.942957	0.000000
H	-3.460081	1.119313	-0.892557
H	-2.311986	2.154740	0.000000
H	-3.460081	1.119313	0.892557

25
TS-2-2-a el energy= -716.620908479

Mn	1.977425	2.318140	0.943203
O	2.822500	1.044964	-1.557912
O	0.111184	3.972731	-0.650479
O	0.172123	0.043048	1.494901
C	3.591616	1.603691	2.229674
C	3.982231	2.706558	1.406287
C	3.183427	3.876776	1.522880
C	2.209334	3.846471	2.666718
C	2.826010	3.277814	3.962974

C	3.296765	1.820604	3.722480
C	2.354193	0.740712	4.323836
C	2.480364	1.547535	-0.558677
C	0.839037	3.322225	-0.009160
C	0.889668	0.939629	1.279208
H	4.030669	0.626223	1.989228
H	1.686695	4.810035	2.787901
H	4.716528	2.610696	0.596690
H	3.365267	4.790385	0.950285
H	1.303344	3.121629	2.453334
H	2.093969	3.323910	4.789509
H	3.675501	3.925976	4.245923
H	4.279097	1.713050	4.229021
H	2.897406	0.074603	5.017244
H	1.901565	0.106601	3.544386
H	1.525103	1.194510	4.896496

25

TS-2-2-b el energy= -716.608085252

Mn	1.840049	2.363714	1.012976
O	1.490573	-0.487387	0.417929
O	1.553198	3.268081	-1.764186
O	-1.076650	2.616028	1.332677
C	3.596881	1.538899	2.113323
C	3.984408	2.510393	1.142724
C	3.359935	3.782659	1.263256
C	2.612055	3.996177	2.545941
C	3.368606	3.427056	3.770043
C	3.494709	1.897782	3.600654
C	2.381647	1.123369	4.326185
C	1.604640	0.648474	0.678403
C	1.647409	2.919488	-0.651628
C	0.082328	2.508335	1.196697
H	3.892635	0.499519	1.918202
H	2.259925	5.035485	2.650007
H	4.547789	2.242013	0.241336
H	3.570833	4.613584	0.583742
H	1.614919	3.387905	2.567458
H	2.831933	3.675551	4.703792
H	4.363985	3.904546	3.821419
H	4.460843	1.577482	4.047549
H	2.465653	0.037725	4.137096
H	1.381840	1.437502	3.974289
H	2.437683	1.288295	5.418026

25
TS-3-3-a el energy= -716.615353074

Mn	2.084980	2.230656	1.154497
O	2.424507	-0.675691	0.704749
O	2.459187	2.628413	-1.713523
O	-0.837812	2.460670	0.744604
C	4.041657	2.525371	2.017501
C	3.497994	3.742753	1.520639
C	2.248857	4.207296	2.014157
C	1.845552	3.859600	3.438803
C	2.252595	2.398827	3.719560
C	3.750292	2.103869	3.457441
C	4.111594	0.623474	3.743507
C	2.288828	0.468707	0.897288
C	2.304946	2.465844	-0.564467
C	0.313747	2.361462	0.913959
H	4.949268	2.128266	1.546467
H	2.327946	4.544153	4.169091
H	3.915700	4.186587	0.607300
H	1.792012	5.085190	1.543378
H	0.753949	3.974174	3.569123
H	1.625201	1.700624	3.081073
H	1.959373	2.085647	4.739660
H	4.347117	2.748387	4.139099
H	4.896556	0.539636	4.514675
H	4.476744	0.110334	2.837124
H	3.236684	0.052547	4.105250

25
TS-3-3-b el energy= -716.598503642

Mn	2.171906	2.206662	1.041142
O	3.477202	0.147475	-0.576907
O	0.910143	3.609520	-1.195328
O	-0.207115	0.486962	1.205162
C	4.087143	2.288574	2.166559
C	3.823912	3.523025	1.520770
C	2.629258	4.222320	1.823744
C	2.021282	4.089355	3.213346
C	2.179982	2.660645	3.784640
C	3.590023	2.047867	3.590981
C	3.614375	0.561104	3.973850
C	2.942680	0.941489	0.098469
C	1.380916	3.050398	-0.279846
C	0.743658	1.169348	1.121157
H	4.959594	1.706889	1.843794

H	2.520203	4.824465	3.881506
H	4.376676	3.778453	0.606389
H	2.390124	5.126917	1.253134
H	0.951882	4.366102	3.194496
H	1.434769	1.986494	3.301209
H	1.906258	2.645176	4.856546
H	4.297791	2.582229	4.264286
H	4.629785	0.138997	3.863980
H	2.935325	-0.021682	3.324369
H	3.299701	0.418011	5.023505

25
TS-4-4-a el energy= -716.608468531

Mn	1.262920	-0.009226	0.000000
O	2.530266	1.381838	-2.288813
O	3.578834	-1.778251	0.000000
O	2.530266	1.381838	2.288813
C	-1.462003	-0.266861	-1.278152
C	-0.182542	-1.097450	-1.215735
C	0.128099	-1.767389	0.000000
C	-0.182542	-1.097450	1.215735
C	-1.462003	-0.266861	1.278152
C	-1.710056	0.571874	0.000000
C	-0.799666	1.821317	0.000000
C	2.017222	0.853713	-1.381679
C	2.647680	-1.066677	0.000000
C	2.017222	0.853713	1.381679
H	-1.453514	0.400455	-2.160547
H	-2.309425	-0.968946	-1.432573
H	0.148172	-1.552705	-2.157041
H	0.750700	-2.671429	0.000000
H	0.148172	-1.552705	2.157041
H	-1.453514	0.400455	2.160547
H	-2.309425	-0.968946	1.432573
H	-2.767763	0.895927	0.000000
H	-0.152136	1.865818	-0.899776
H	-0.152136	1.865818	0.899776
H	-1.362826	2.772129	0.000000

25
TS-4-4-b el energy= -716.591914824

Mn	1.330213	-0.000023	0.012584
O	3.052853	0.881240	2.209763
O	3.088817	0.908246	-2.143571
O	2.922763	-2.482934	0.018265

C	-1.457933	0.231137	1.286102
C	-0.214538	1.107225	1.205214
C	0.093689	1.761869	-0.015455
C	-0.225324	1.089928	-1.222996
C	-1.483246	0.232468	-1.283076
C	-1.755519	-0.580749	0.004323
C	-0.932360	-1.867763	-0.017426
C	2.353227	0.497545	1.351716
C	2.376561	0.513213	-1.301207
C	2.276250	-1.504911	0.011968
H	-1.393868	-0.455760	2.150794
H	-2.310527	0.910540	1.505647
H	0.087545	1.592133	2.141252
H	0.758779	2.634419	-0.023894
H	0.084502	1.551419	-2.168206
H	-1.445981	-0.452596	-2.150940
H	-2.331248	0.923444	-1.482204
H	-2.826225	-0.865839	0.018240
H	-1.028078	-2.452788	0.914473
H	0.168497	-1.658930	-0.143466
H	-1.188850	-2.512532	-0.877902

25

TS-3-5 el energy= -716.563089292

Mn	-0.464232	0.007595	-0.209595
O	-1.399166	2.560111	0.976726
O	-3.302943	-0.740698	-0.559106
O	0.368259	1.569612	-2.570766
C	1.013835	0.115890	1.402623
C	0.009677	-0.860059	1.684811
C	-0.201865	-1.934789	0.790399
C	0.910811	-2.331036	-0.166860
C	1.418815	-0.961614	-0.608353
C	2.186893	-0.245305	0.502089
C	2.976622	0.982555	0.038452
C	-1.035887	1.541243	0.542171
C	-2.191716	-0.420235	-0.405371
C	0.050241	0.989835	-1.609816
H	1.089351	0.986402	2.068966
H	1.685469	-2.976371	0.314436
H	-0.728816	-0.660020	2.471209
H	-1.045288	-2.611412	0.977315
H	0.493027	-2.895130	-1.018784
H	-0.804016	-0.836006	-1.489918
H	1.863337	-0.910046	-1.613344

H	2.901759	-0.925320	1.028835
H	3.449025	1.496468	0.896012
H	2.322773	1.710610	-0.474276
H	3.778507	0.687920	-0.662214

25
TS-4-6 el energy= -716.571674841

Mn	1.190422	-0.002182	0.000000
O	2.215666	0.934842	-2.609970
O	3.438601	-1.928248	0.000000
O	2.215666	0.934842	2.609970
C	-1.584319	-0.075243	-1.270205
C	-0.386116	-1.003891	-1.214435
C	-0.086940	-1.680068	0.000000
C	-0.386116	-1.003891	1.214435
C	-1.584319	-0.075243	1.270205
C	-1.611328	0.784562	0.000000
C	-0.323768	1.603878	0.000000
C	1.812072	0.540927	-1.589201
C	2.526967	-1.197360	0.000000
C	1.812072	0.540927	1.589201
H	-1.526380	0.578442	-2.160819
H	-2.509981	-0.680574	-1.381945
H	-0.074551	-1.487347	-2.149744
H	0.460366	-2.629695	0.000000
H	-0.074551	-1.487347	2.149744
H	-1.526380	0.578442	2.160819
H	-2.509981	-0.680574	1.381945
H	-2.509752	1.439780	0.000000
H	-0.251708	2.248312	-0.894929
H	1.526070	1.498397	0.000000
H	-0.251708	2.248312	0.894929

25
TS-1-2 el energy= -716.603863047

Mn	-0.505663	-0.088418	-0.112176
O	-0.512498	2.631212	-1.248420
O	-3.045243	0.410744	1.341004
O	-2.135442	-1.571785	-2.084041
C	1.189790	0.628316	1.090092
C	0.397676	-0.337418	1.771639
C	0.256034	-1.609940	1.115814
C	0.961882	-1.711429	-0.117952
C	2.375755	-1.159927	-0.237029
C	2.503181	0.240453	0.418228

C	2.998070	1.304442	-0.572824
C	-0.541415	1.557920	-0.793064
C	-2.027017	0.209676	0.809313
C	-1.508816	-0.979573	-1.299943
H	0.437993	-0.062981	-1.370364
H	1.150147	1.654806	1.480653
H	-0.176378	-0.098046	2.673848
H	-0.447463	-2.369088	1.471791
H	0.730735	-2.572580	-0.758644
H	2.661789	-1.111231	-1.303680
H	3.071214	-1.882569	0.238316
H	3.255711	0.183006	1.234985
H	4.002704	1.044292	-0.953376
H	3.063028	2.300406	-0.096730
H	2.314899	1.386678	-1.437777

25

TS-2-3 el energy= -716.607568768

Mn	2.031319	2.391755	0.882930
O	2.661852	0.844698	-1.504680
O	0.378939	4.156163	-0.834920
O	-0.012500	0.392445	1.638966
C	3.546979	1.580508	2.181323
C	4.061281	2.603927	1.324709
C	3.431568	3.866984	1.383057
C	2.569032	4.253558	2.590116
C	2.450710	3.242480	3.776766
C	3.267631	1.935615	3.641462
C	2.605698	0.785972	4.414389
C	2.393624	1.459926	-0.544600
C	1.022394	3.477605	-0.134370
C	0.793369	1.180723	1.330563
H	3.904616	0.556806	2.008743
H	2.943811	5.233054	2.947035
H	4.754254	2.381482	0.503135
H	3.743060	4.680379	0.719187
H	1.539104	4.499406	2.236349
H	1.385048	2.964799	3.886519
H	2.724218	3.746343	4.721718
H	4.266487	2.109585	4.099791
H	3.233010	-0.123886	4.391113
H	1.622367	0.529411	3.980135
H	2.450182	1.059060	5.474591

25

TS-1-4   el energy= -716.606968990

Mn	0.563841	-0.025812	0.017282
O	0.996123	-2.295731	-1.832561
O	3.235799	-0.425764	1.102123
O	1.612348	2.196198	-1.638958
C	-2.040907	-1.202433	0.558509
C	-0.701019	-1.166836	1.283682
C	-0.265163	0.022599	1.933215
C	-0.531204	1.257418	1.288401
C	-1.773547	1.434969	0.416939
C	-2.610248	0.161884	0.047734
C	-2.840556	0.108780	-1.473048
C	0.832337	-1.393269	-1.108831
C	2.166159	-0.266498	0.650204
C	1.193281	1.318088	-0.992169
H	-1.987533	-1.917277	-0.285108
H	-2.770052	-1.650801	1.266490
H	-0.363099	-2.129178	1.686444
H	0.469404	-0.022786	2.747053
H	-0.101458	2.178129	1.698866
H	-1.499448	1.969223	-0.516451
H	-2.430877	2.152063	0.951635
H	-3.600246	0.288697	0.523680
H	-3.502452	-0.731988	-1.750574
H	-1.880732	-0.029463	-2.011073
H	-3.300163	1.042792	-1.845989

25

TS-1-7   el energy= -716.599703339

Mn	-0.773899	-2.942555	0.284077
O	1.158863	-3.172947	-1.952930
O	-2.755768	-2.516736	-1.870873
O	-2.328505	-5.276934	1.192290
C	0.629047	-2.571416	1.935553
C	0.364900	-1.404507	1.152712
C	-1.011238	-1.031875	1.065947
C	-1.908127	-1.885585	1.792782
C	-1.551210	-2.386893	3.183335
C	-0.057168	-2.783025	3.281302
C	0.140867	-4.209788	3.815454
C	0.404330	-3.054861	-1.071314
C	-1.979814	-2.676526	-1.014307
C	-1.725255	-4.340541	0.840164
H	0.097750	-4.223014	0.412329
H	1.643454	-2.987872	1.859057

H	1.132587	-0.899397	0.555616
H	-1.351903	-0.223374	0.410208
H	-2.980551	-1.751356	1.596607
H	-2.192355	-3.252710	3.434362
H	-1.800607	-1.598607	3.924461
H	0.448439	-2.095065	3.994620
H	1.214214	-4.466798	3.884282
H	-0.345913	-4.951472	3.157180
H	-0.296266	-4.313069	4.825401

25
TS-7-8 el energy= -716.583203875

Mn	0.129071	0.024547	0.124659
O	2.630905	1.592186	-0.032139
O	-0.917240	1.448252	2.487550
O	2.080625	-1.704805	1.536623
C	-1.709328	0.423018	-0.945256
C	-0.827640	-0.473613	-1.658229
C	-0.500783	-1.661934	-0.954864
C	-1.073327	-1.738421	0.377222
C	-2.516780	-1.342283	0.635802
C	-2.949255	-0.169764	-0.274293
C	-3.797710	0.877895	0.460394
C	1.602541	1.005837	-0.050884
C	-0.511312	0.900452	1.540007
C	1.318584	-1.024686	0.976225
H	0.627886	1.250420	-0.845790
H	-1.839311	1.426350	-1.374844
H	-0.350017	-0.224581	-2.613938
H	0.216307	-2.392737	-1.343755
H	-0.700429	-2.552996	1.012874
H	-2.627227	-1.059563	1.700216
H	-3.174180	-2.225723	0.485992
H	-3.577282	-0.580907	-1.095016
H	-4.705818	0.413055	0.886582
H	-4.124225	1.680671	-0.226339
H	-3.236697	1.344625	1.288514

25
TS-8-9 el energy= -716.583758176

Mn	-0.030268	0.485601	0.529306
O	-0.190284	3.355431	-0.190823
O	-1.548263	0.562555	3.057695
O	2.464087	1.572750	1.681310
C	-1.640489	0.476938	-0.906901

C	-0.408949	-0.062903	-1.453700
C	0.087804	-1.181495	-0.733721
C	-0.687128	-1.571778	0.416824
C	-2.204139	-1.635158	0.336145
C	-2.771805	-0.464966	-0.503052
C	-3.921704	0.269238	0.202495
C	-0.122164	2.226036	0.088209
C	-0.944618	0.540435	2.058220
C	1.509737	1.052647	1.211622
H	1.383767	-0.206865	1.001033
H	-1.982720	1.424701	-1.346332
H	0.135223	0.386211	-2.290946
H	1.070936	-1.612503	-0.960326
H	-0.217505	-2.292327	1.099683
H	-2.625229	-1.609982	1.358487
H	-2.509116	-2.611787	-0.095310
H	-3.178084	-0.872526	-1.455590
H	-4.748294	-0.428981	0.428182
H	-4.328374	1.080687	-0.429093
H	-3.584040	0.714832	1.154856

25

TS-2-9 el energy= -716.599009254

Mn	-0.054301	0.621405	0.550312
O	0.592260	3.439783	-0.084250
O	-1.941314	1.572131	2.607161
O	2.817615	0.452422	1.268163
C	-1.745507	0.525805	-0.807261
C	-0.495866	0.154201	-1.413494
C	0.135229	-0.975943	-0.807640
C	-0.532524	-1.510264	0.339279
C	-2.043598	-1.651986	0.396008
C	-2.748795	-0.574887	-0.456657
C	-4.029873	-0.042383	0.200451
C	0.327865	2.328668	0.152278
C	-1.218650	1.200723	1.767663
C	1.693031	0.511058	0.964236
H	0.122997	-0.100086	1.914477
H	-2.179084	1.486833	-1.117368
H	-0.017715	0.717538	-2.222267
H	1.133705	-1.307268	-1.114751
H	0.033269	-2.240536	0.933584
H	-2.375546	-1.578164	1.448983
H	-2.328788	-2.670965	0.057164
H	-3.043120	-1.035209	-1.425151

H	-4.740332	-0.867469	0.391730
H	-4.538459	0.693138	-0.449835
H	-3.814119	0.448246	1.165462
